# Genomic insights of *mcr-1* harboring *Escherichia coli* by geographical region and a One-Health perspective

**DOI:** 10.3389/fmicb.2022.1032753

**Published:** 2023-01-16

**Authors:** William Calero-Cáceres, Kerly Rodríguez, Anabell Medina, Jennifer Medina, Nimer Ortuño-Gutiérrez, Temmy Sunyoto, Cícero Armídio Gomes Dias, Carlos Bastidas-Caldes, Maria Soledad Ramírez, Anthony David Harries

**Affiliations:** ^1^UTA RAM One Health, Department of Food and Biotechnology Science and Engineering, Universidad Técnica de Ambato, Ambato, Ecuador; ^2^Bacteriophage Research Association, Ambato, Ecuador; ^3^Damien Foundation, Brussels, Belgium; ^4^MSFOCB Luxembourg Operational Research (LuxOR) Unit, Luxembourg, Luxembourg; ^5^Department of Basic Health Sciences, Federal University of Health Sciences of Porto Alegre (UFCSPA), Porto Alegre, Brazil; ^6^One Health Research Group, Biotecnología, Facultad de Ingeniería y Ciencias Aplicadas (FICA), Universidad de las Américas (UDLA), Quito, Ecuador; ^7^Department of Biological Science, College of Natural Sciences and Mathematics, California State University Fullerton, Fullerton, CA, United States; ^8^International Union Against Tuberculosis and Lung Disease, Paris, France; ^9^London School of Hygiene and Tropical Medicine, London, United Kingdom

**Keywords:** One Health, antibiotic resistance genes, *Escherichia coli*, whole-genome sequencing, colistin resistance, *mcr-1*

## Abstract

The importance of the One Health concept in attempting to deal with the increasing levels of multidrug-resistant bacteria in both human and animal health is a challenge for the scientific community, policymakers, and the industry. The discovery of the plasmid-borne mobile colistin resistance (*mcr*) in 2015 poses a significant threat because of the ability of these plasmids to move between different bacterial species through horizontal gene transfer. In light of these findings, the World Health Organization (WHO) recommends that countries implement surveillance strategies to detect the presence of plasmid-mediated colistin-resistant microorganisms and take suitable measures to control and prevent their dissemination. Seven years later, ten different variants of the *mcr* gene (*mcr-1* to *mcr-10*) have been detected worldwide in bacteria isolated from humans, animals, foods, the environment, and farms. However, the possible transmission mechanisms of the *mcr* gene among isolates from different geographical origins and sources are largely unknown. This article presents an analysis of whole-genome sequences of *Escherichia coli* that harbor *mcr-1* gene from different origins (human, animal, food, or environment) and geographical location, to identify specific patterns related to virulence genes, plasmid content and antibiotic resistance genes, as well as their phylogeny and their distribution with their origin. In general, *E. coli* isolates that harbor *mcr-1* showed a wide plethora of ARGs. Regarding the plasmid content, the highest concentration of plasmids was found in animal samples. In turn, Asia was the continent that led with the largest diversity and occurrence of these plasmids. Finally, about virulence genes, *terC, gad,* and *traT* represent the most frequent virulence genes detected. These findings highlight the relevance of analyzing the environmental settings as an integrative part of the surveillance programs to understand the origins and dissemination of antimicrobial resistance.

## Introduction

Over the last two decades, the evolution and dissemination of new antibiotic resistance determinants has led to one of the most critical health scenarios worldwide. According to the United Nations and the World Bank, antimicrobial resistance (AMR) will cause, in the worst scenario, 10 million deaths every year and a loss of 3.8 percent of the annual global gross domestic product (GDP) by 2050 (World Bank, 2017; IACG, 2019). A recently published systematic analysis on the worldwide burden of bacterial AMR estimated that in 2019 there were nearly 5 million deaths associated with AMR in that year alone, and 1.3 million deaths were directly attributable to bacterial AMR (Murray et al., 2022). The Global Burden of Diseases, Injuries and Risk Factors Study (GBD) estimates that in 2019 out of 13.7 million deaths, the leading pathogen responsible for global deaths was *Staphylococcus aureus*, followed closely by *Escherichia coli*, *Streptococcus pneumoniae* and *Klebsiella pneumoniae* (GBD, 2019, Antimicrobial Resistance Collaborators, 2022).

Although bacterial AMR represents a threat to humankind, pharmaceutical research and development have failed to identify and deploy new antibiotics that can reduce and minimize this substantial danger (Tacconelli et al., 2017). In this context, the dependence on antibiotics of last resort to treat the increasing levels of multidrug-resistant bacteria in both human and animal health is a challenge for the scientific community, policymakers, and the industry, and hence the importance of the One Health concept in attempting to deal with this global problem (Mendelson et al., 2018). Colistin (polymyxin E) is considered one of the crucial last-resort antibiotics and is regarded as an essential option for use against infections caused by multidrug-resistant Gram-negative bacteria (Falagas et al., 2005; Kaye et al., 2016). Unfortunately, colistin is commonly used in the animal world for treating infections and as a growth promoter in animal feeds. These practices have contributed to widescale and rapid global resistance to this antibiotic (Elbediwi et al., 2019).

Traditionally, colistin resistance mechanisms were triggered by chromosomal mutations (Kim et al., 2019). However, the discovery of the plasmid-borne mobile colistin resistance mechanism in 2015, called *mcr*, poses a significant threat because of the ability of these plasmids to move between different bacterial species through horizontal gene transfer (Liu et al., 2015). In light of these findings, the World Health Organization (WHO) recommends that countries implement surveillance strategies to detect the presence of plasmid-mediated colistin-resistant microorganisms and take suitable measures to control and prevent their dissemination (PAHO/WHO, 2016).

Since 2015, the *mcr* gene has been reported in the chromosomal or plasmid content in some bacterial species, mainly the Enterobacterales (Zhang et al., 2021). Nowadays, the *mcr* gene is most frequently harbored with *E. coli* (Forde et al., 2018). At present, ten different variants of the *mcr* gene (*mcr-1* to *mcr-10*) have been detected worldwide in bacteria isolated from humans, animals, foods, the environment, and farms (Dadashi et al., 2021; Hussein et al., 2021). However, the possible transmission mechanisms of the *mcr* gene among isolates from different geographical origins and sources are largely unknown.

The democratization of whole-genome sequencing (WGS) techniques and the generation of bioinformatic platforms that allow a deep characterization of pathogens can potentially transform epidemiological surveillance and disease control systems worldwide (NIHR Global Health Research Unit on Genomic Surveillance of AMR, 2020; Ferdinand et al., 2021; Kovac et al., 2021). Using these new technologies, the aim of this study was to re-analyze *mcr-1* harboring *E. coli* WGS sequences submitted to the greater public genomic databases [such as the National Center for Biotechnology Information (NCBI) and the European Nucleotide Archive (ENA)] to identify specific patterns related to virulence genes, plasmid content and antibiotic resistance genes, as well as their phylogeny and their distribution with their origin (human, animal, food or environment) and geographical location.

## Materials and methods

### Sequences

One hundred and twenty-three WGS sequences of *mcr-1* harboring *E. coli* were obtained from NCBI and ENA databases from 26 countries from four continents (Asia, Africa, the Americas, and Europe). The description of the sequences (years, country of origin, and NCBI/ENA number) are included in Supplementary Table S1. Briefly, 42 sequences were retrieved from the Americas, 32 from Europe, 10 from South and East Asia, 15 from Western Asia, 22 from Southeast Asia, and two from Africa. According to their origin, 55 were from animals, 47 were from humans, 7 were from feeds, and 14 were from environmental samples. In addition, the draft genome assembly quality was evaluated using CheckM v1.0.18.

### Bioinformatic analyses

Draft genomes were analyzed using the tools from the Center for Genomic Epidemiology of the Technical University of Denmark. Multilocus sequence types (MLSTs) were determined using MLST v2.0 (Larsen et al., 2012). The presence of plasmids was identified using PlasmidFinder (Carattoli and Hasman, 2020). The prediction of bacterial pathogenicity was evaluated using PathogenFinder v1.1 (Cosentino et al., 2013). The detection of *E. coli* virulence genes was carried out using VirulenceFinder v2.0 (Tetzschner et al., 2020). The identification of acquired genes and/or chromosomal mutations mediating antimicrobial resistance was done using ResFinder 4.1 (Bortolaia et al., 2020). The core-genome multilocus sequence typing phylogeny was carried out using the Galaxy Sciensano platform (Bogaerts et al., 2019). The obtained Newick file was analyzed and annotated using iTOL platform (Letunic and Bork, 2021).

### Statistical analysis

The distribution of the genotypes between hosts and location was calculated using a one-way analysis of variance (ANOVA). Tukey’s multiple-comparison test was performed *post hoc* for pairwise comparisons between groups, and *p*-values < 0.05 were considered significant. The data analysis for this paper was generated using the Real Statistics Resource Pack software (Release 7.6). Copyright (2013–2021) Charles Zaiontz.^1^

### Ethics statement

This work does not involve the use of human subjects or animal experiments.

## Results and discussion

**Figure 1:**
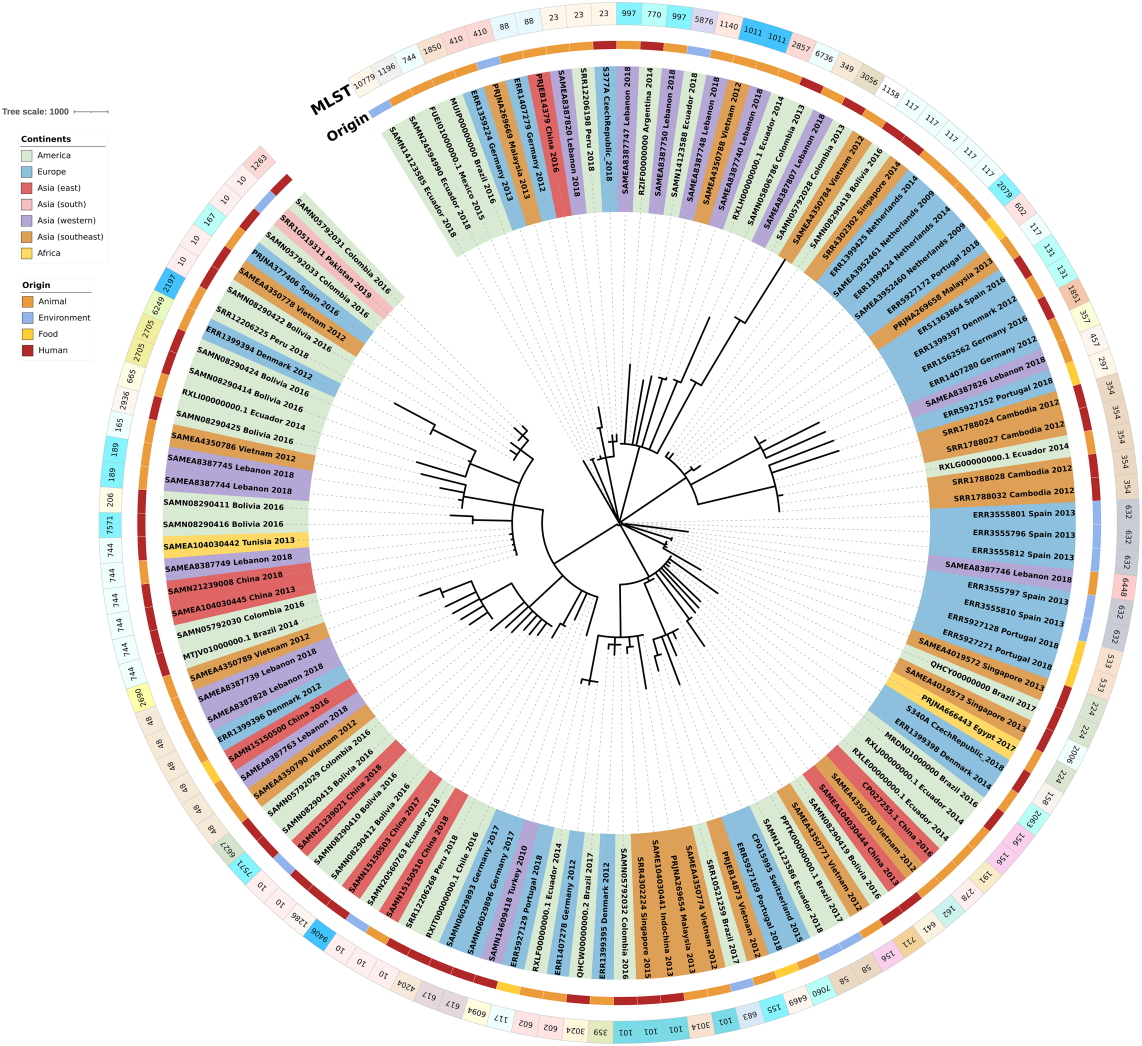
Phylogenetic tree of the core-genome multilocus sequence type allelic profiles of *mcr-1* harboring *Escherichia coli* isolates evaluated in this study. Clusters are colored and labeled according to their continent of origin and the source of isolation.

**Figure 2:**
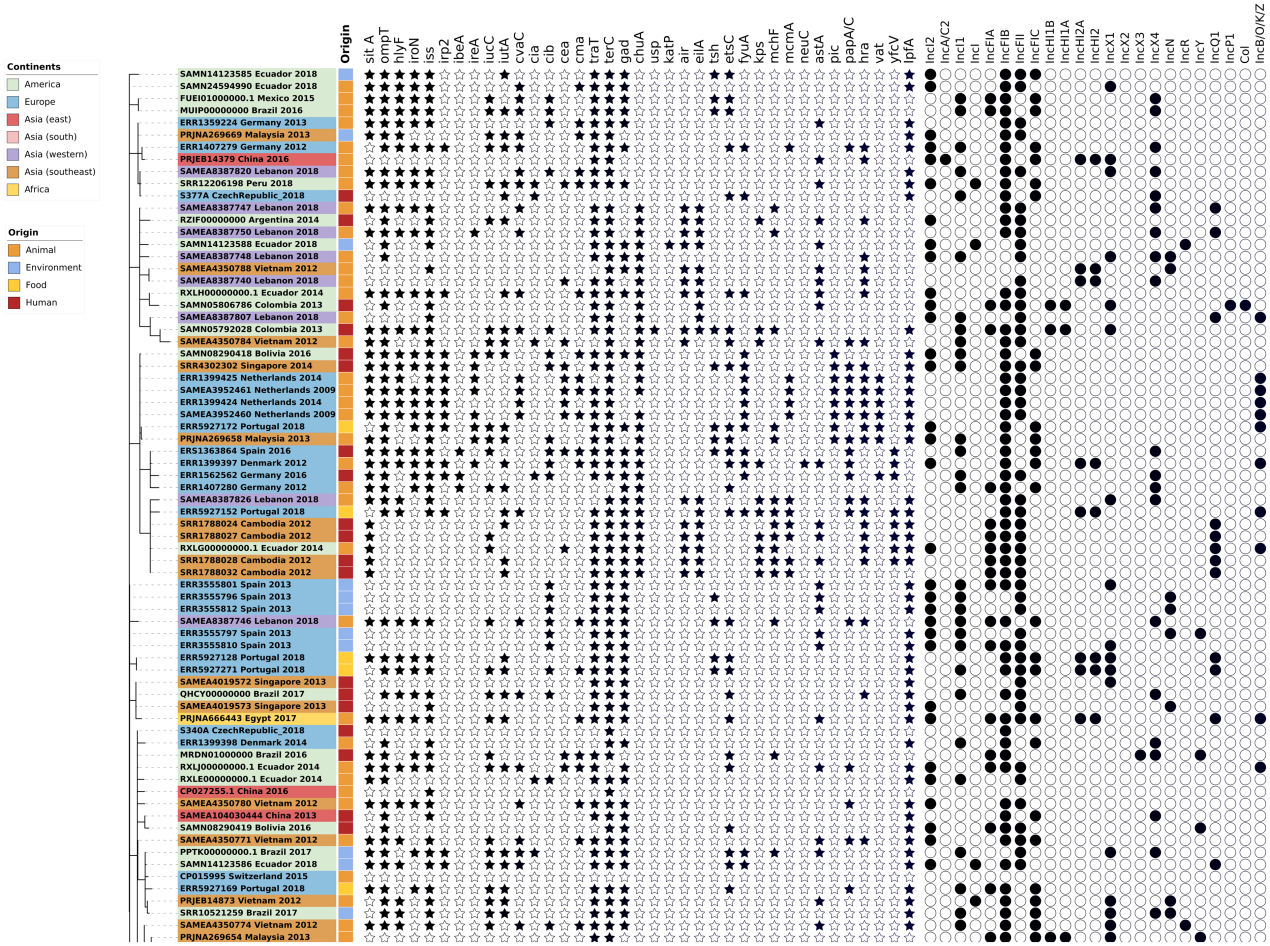
Virulence genes and plasmid content of the evaluated *E. coli* sequences (Part one). The source of the isolates is specified by different colors in the genomic cg-MLST tree branches. The presence and absence of virulence genes and plasmid incompatibility groups are indicated by star and circle symbols, respectively.

**Figure 3:**
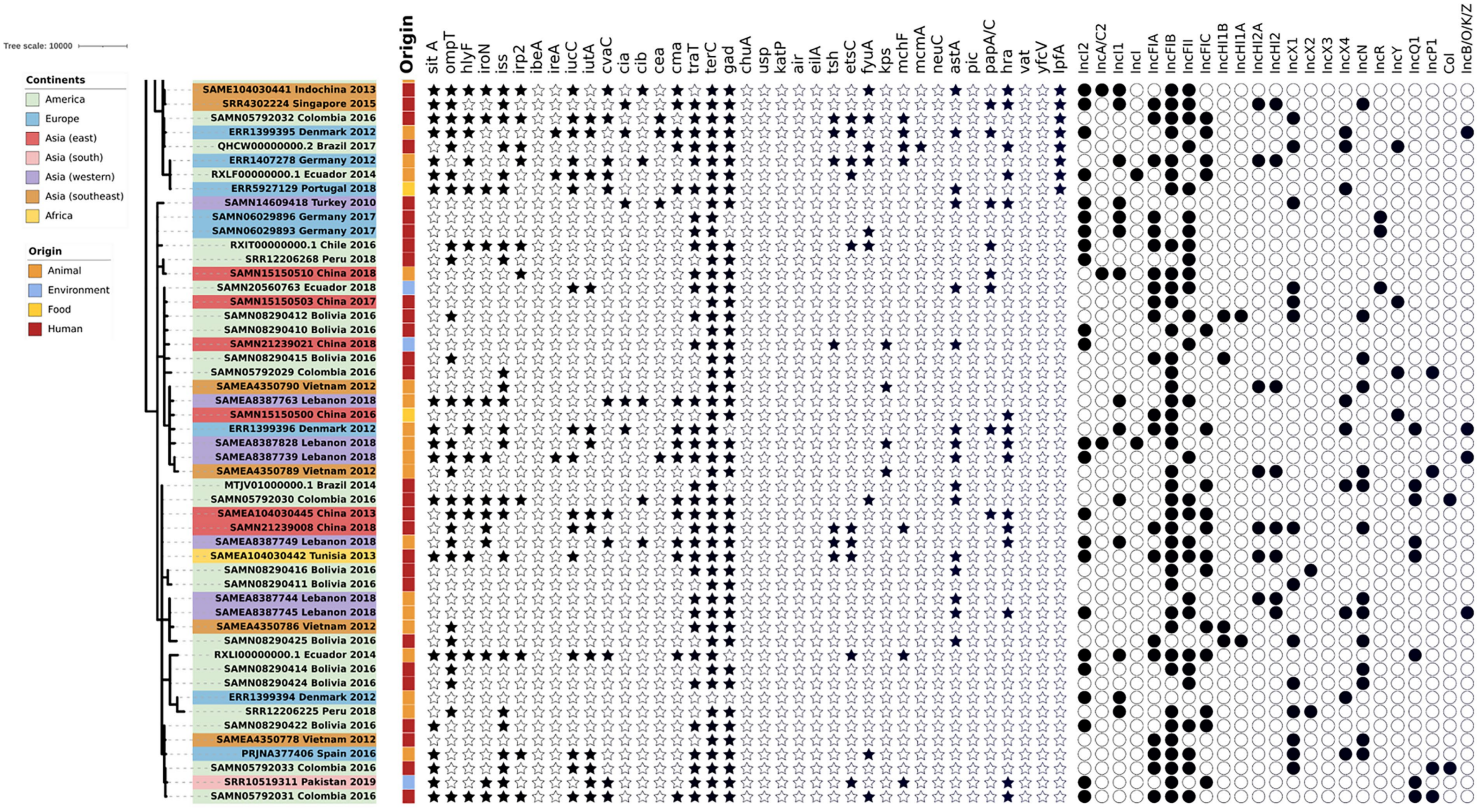
Virulence genes and plasmid content of the evaluated *E. coli* sequences (Part two). The source of the isolates is specified by different colors in the genomic cg-MLST tree branches. The presence and absence of virulence genes and plasmid incompatibility groups are indicated by star and circle symbols, respectively.

**Figure 4:**
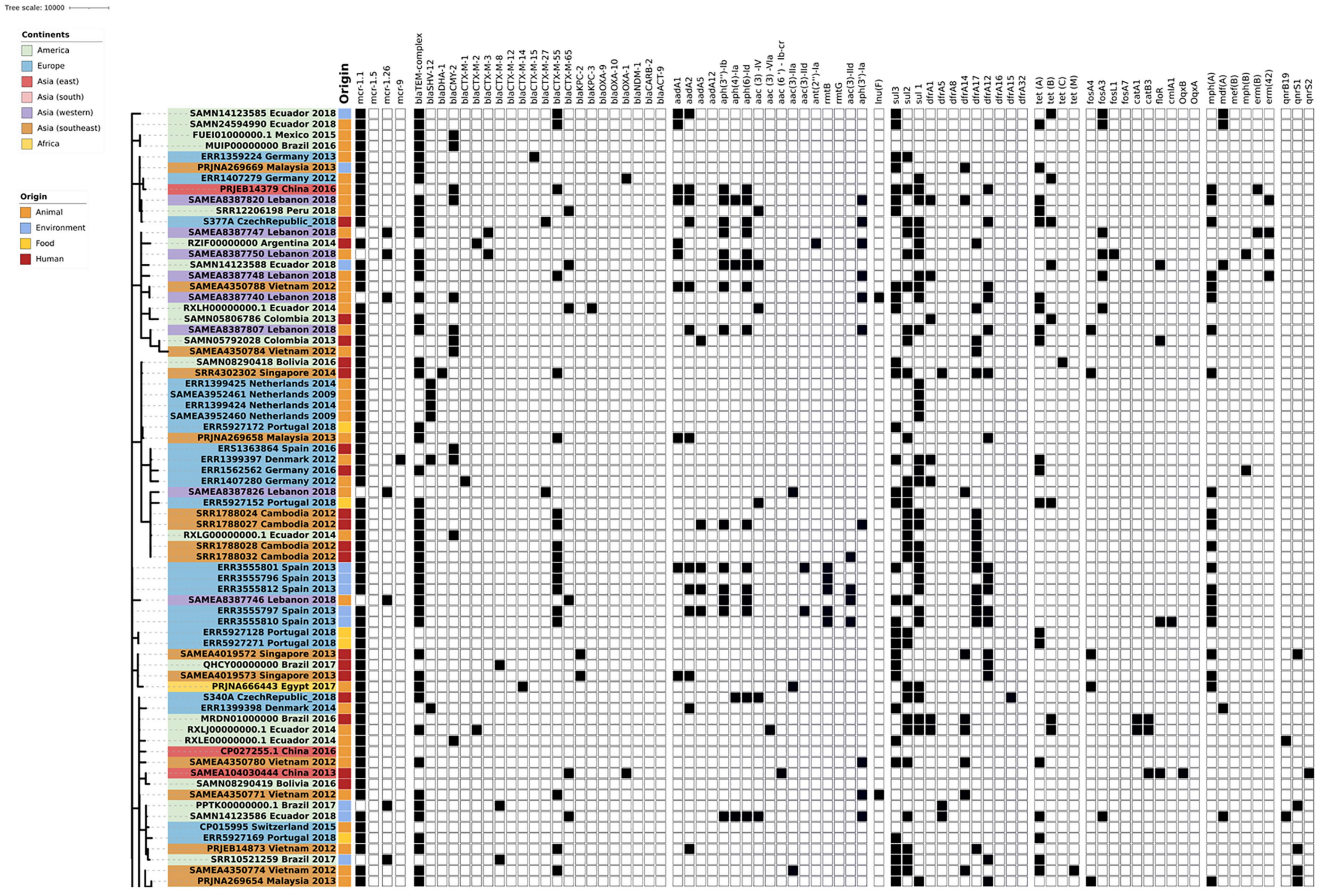
Antibiotic resistance genes content of the evaluated *E. coli* sequences (Part one). The source of the isolates is specified by different colors in the genomic cg-MLST tree branches. Squares indicate the presence (black) and absence (white) of antibiotic resistance genes.

**Figure 5:**
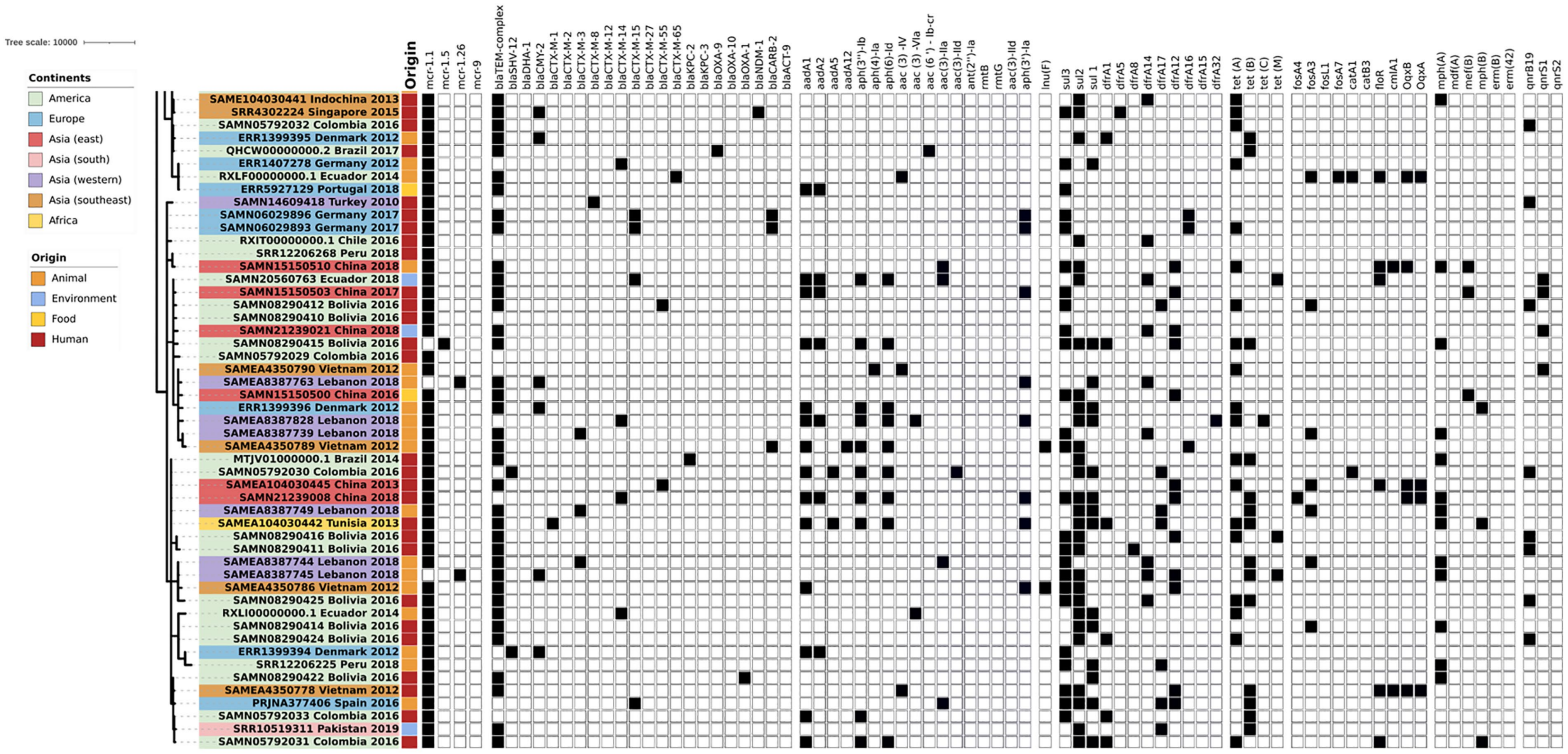
Antibiotic resistance genes content of the evaluated *E. coli* sequences (Part two). The source of the isolates is specified by different colors in the genomic cg-MLST tree branches. Squares indicate the presence (black) and absence (white) of antibiotic resistance genes.

The phylogenetic tree of the core-genome multilocus sequence type allelic profiles of *mcr-1* harboring *E. coli* isolates evaluated in this study is shown in Figure 1, which includes the MLST profile of the isolates, the origin of isolation, and the continent. The name of the isolate corresponds to their NCBI, ENA, or internal database, followed by the country and the year of isolation. The virulence genes and plasmid content of the evaluated *E. coli* sequences are shown in Figures 2, 3. The antibiotic-resistance genes are indicated in Figures 4, 5. A comparison of the topologies of the cgMLST-phylogenetic tree reveals that the evolutionary relationships among *mcr-1* harboring *E. coli* were not entirely correlated with the origin of the samples (animal, environmental, human, and food/feed) and the geographical precedence.

### Virulence

The presence of virulence genes stratified by the origin of the samples (environmental, human, food and animal) was generally homogeneous (*p* < 0.05, one-way ANOVA with Tukey’s multiple-comparison test). Globally, one of the most frequent virulence genes detected was the virulence factor *terC* (resistance to tellurium), which had an incidence of 96%. However, even though the *Ter* operon role was associated with pathogenicity and stress response, the biochemical mechanisms of this association are still unclear (Turkovicova et al., 2016). The resistance followed by the *gad* factor (glutamate decarboxylase system) had an incidence of 77%, and *traT* (serum resistance in *E. coli*) had an incidence of 76% of the evaluated genomes. The presence of *gad* genes was suggested as a prescreening marker for the detection of pathogenic *E. coli* groups and this was applied to evaluate the load of pathogens in environmental samples (Grant et al., 2001; Hoorzook and Barnard, 2021). Both genes, *gad* and *traT* were often detected in clinical and animal isolates of *E. coli* worldwide (Firoozeh et al., 2014; Huang et al., 2021).

The *sitA* virulence vector (prevents unproductive conjugation) was recorded in all of the animal samples from 2014 and 2018; *sitA* was applied as an excellent genetic marker for the identification of avian pathogenic *E. coli*, considering their significantly difference in the expression on pathogenic vs. non-pathogenic strains (Schouler et al., 2012). In addition, this gene was frequently detected in highly-pathogenic extra-intestinal *E. coli* (ExPEC) isolates worldwide (Bakhshi et al., 2020). In the *E. coli* isolates from environmental and food/feed samples, the *lpfA* gene (long polar fimbriae associated with adherence) was detected with considerable frequency (71 and 86% of the analyzed genomes) in comparison with isolates from human and animal origin. *lpfA* was commonly associated with enteropathogenic *E. coli* (Ross et al., 2015; Zhou et al., 2021). In a recent study carried out in Brazil, there was a statistically significant increased presence of the *lpfA* gene (*p* < 0.05) in *E. coli* from agricultural soils in comparison with the isolates from non-agriculture origin (Furlan and Stehling, 2021), highlighting the role of animal manure as potential source of virulence genes.

Another virulence gene frequently detected in environmental samples was *iutA* (ferric aerobactin receptor). The presence of this gene was significatively higher in extraintestinal pathogenic *E. coli*, and their presence might be a disadvantage for colonization in humans and animals (Ikeda et al., 2021). In contrast, the gene *iss* (serum survival) was highly correlated with isolates from human, animal, and food/feed origin. This gene could influence capsule production in bacteria (Biran et al., 2021). The rest of the virulence genes were distributed in homogenous and lower densities among the isolates from different sample origins. No significant differences were identified in the detection of virulence genes according to their geographical origin (*p* > 0.05; one-way ANOVA with Tukey’s multiple-comparison test).

### Antibiotic resistance genes

The *mcr-1-*harboring *E. coli* commonly show different antibiotic resistance patterns, and the detection of multiresistant genotypes in these isolates increases their epidemiological relevance significantly. Among the analyzed genomes, the variant *mcr-1.1* is the most frequently detected globally. In western Asia, the variant *mcr-1.26* from different STs was detected in avian isolates from Lebanon. The same variant was detected in isolates from highly polluted water samples from Brazil that included highly-pathogenic STs (Furlan et al., 2020). The variant *mcr-1.5* was detected in Bolivian *E. coli* isolate of human origin (NCBI Accession SAMN08290415). Nowadays, no additional information about this isolate has been published.

Interestingly, one isolate from animal origin in Denmark (NCBI run ERR1399397, NCBI accession PRJEB13885, ENA submission ERA613496) shows the co-harboring of two mobile colistin resistance genes: *mcr-1.1* and *mcr-9*. Additionally, this isolate encodes the extended-spectrum β-lactamases (ESBL) genes *bla*_SHV_ and *bla*_CMY_. However, no additional information about this isolate was published until now. The co-occurrence of *mcr* gene variants is not frequently detected; however, there are a few recent reports from an *E. coli* isolate of human origin in Thailand (*mcr-2* and *mcr-3*; Phuadraksa et al., 2022). Another study that reports the occurrence of co-harbored *mcr* variants was conducted in Laos, detecting *mcr-3* and *mcr-8* genes inside one isolate from *Klebsiella pneumoniae* (accession number CP035204; Hadjadj et al., 2019). Furthermore, the detection in the same strain of different variants of *mcr* genes poses a threat to the spread of colistin resistance through horizontal and vertical gene transfer.

Most *mcr*-harboring bacteria possess multidrug resistance phenotypes and their subsequent antibiotic-resistance genes (ARGs). No significant differences were identified according to the sample origin (*p* > 0.05; one-way ANOVA). However, significant differences were identified between geographic locations in the detection of ARGs (*p* > 0.05; one-way ANOVA with Tukey’s multiple-comparison test). This was specifically found between the American and African groups and South and East Asian and African groups. Among the vast plethora of ARGs detected *in silico*, the most frequent genes detected were the ESBL genes. *bla*_TEM_ was seen in higher proportions in environmental (93%), human (68%), food/feed (71%), and animal (53%) samples. According to the geographical distribution, this gene was detected worldwide in 50–100% of samples, showing a higher presence in Asian and African isolates. Another ESBL gene with a considerable presence was *bla*_CTX-M_ which has shown extended dissemination in the worldwide community since the 2000s (Chong et al., 2018). Among the CTX-M variants of clinical relevance, *bla*_CTX-M-55_ represented the most frequent gene detected, especially in environmental isolates (43%) and with lower frequency in human and animal samples. The same trend was observed in *bla*_CTX-M-65_ and *bla*_CTX-M-15_, showing higher positive results in environmental samples compared to animal and human isolates. The variants CTX-M-65 and CTX-M-55 showed a familiar presence in isolates from Asia, Europe, and America. Several questions still arise to elucidate these trends that link the environmental sources as a potential reservoir or origin of these CTX-M subtypes and the acceleration of the dissemination in the community.

The increasing resistance to carbapenems (last-resort antibiotic class) represents a severe threat to the healthcare system worldwide (García-Betancur et al., 2021). The WHO global priority pathogen list of antibiotic-resistant bacteria includes *Enterobacterales* carbapenem-resistant as critical (priority 1; WHO, 2017). The carbapenemase enzymes detected in the *mcr-1 E. coli* included KPC, OXA, and NDM in human and animal samples from Singapore, Brazil, Bolivia, Germany, Ecuador, and China. No environmental or feed isolates evaluated show a co-harboring of carbapenemases and *mcr* genes. The dissemination of isolates that harbor *mcr-1* and carbapenemases constitutes a public health issue, although these isolates have been reported in low frequency until now (Dalmolin et al., 2019; Bilal et al., 2021). The increasing detection of isolates that co-harbor critically important ARGs emphasized the importance of prompt surveillance strategies to reduce the dissemination of these highly critical clones worldwide.

Other β-lactamases detected inside the *mcr*-*E. coli*, mainly in human and animal origin isolates, are the *bla*_CARB_ and the *bla*_CMY_ genes. The carbenicillinase resistance gene *bla*_CARB_, earlier known as *bla*_PSE_, is commonly associated with a chromosomal cassette in different bacterial genera, including *Acinetobacter*, *Salmonella*, *Escherichia*, and *Pseudomonas* (Kamolvit et al., 2015; Monte et al., 2020). Furthermore, the presence of *bla*_CMY_ AmpC-β-lactamases are one of the primary mechanisms for resistance to extended-spectrum cephalosporins, and were one of the most common β-lactamases detected in *Enterobacterales* from poultry origin worldwide (Dandachi et al., 2018; Rizi et al., 2020).

Other ARGs commonly detected in *mcr-1* harboring *E. coli* in all the sample types and origins were the resistance genes *sul1, sul2,* and *sul3* that codify resistance to sulfonamides. Considering the prolonged usage of this antibiotic family, the resistance to sulfonamides was widespread in different environments, including pristine locations (Pruden et al., 2012; Lin et al., 2021). Although less commonly found, another group of ARGs frequently detected was the tetracycline-resistance genes *tetA* and *tetB*, which are widely reported on the current clones that circulate worldwide. The extensive use of tetracyclines in veterinary and clinical settings as prophylactics and growth promoters may lead to the selective pressure for developing and disseminating those ARGs (Rose et al., 2016; Samanta and Bandyopadhyay, 2020).

Another antibiotic group extensively used in veterinary practice and which showed a considerable presence of ARGs was the macrolides, which caused a concomitant rise in resistance to these drugs in animal pathogens worldwide (Samanta and Bandyopadhyay, 2020). The ARG *mph*A (macrolide 2′-phosphotransferase I) was detected in the 28% of the evaluated isolates, showing a homogeneous distribution in animal, human and environmental samples. However, a higher presence of this gene was observed in the isolates of southeast and western Asia compared to America and Europe. In addition, the presence of acquired ARGs to the trimethoprim gene *dfrA* (dihydrofolate reductase) variants was considerably higher in Asian countries compared with European and American countries. In *Enterobacterales*, these genes are usually part of mobile gene cassettes associated with integrons (Partridge et al., 2009). This ARG is widespread in Gram-negative bacteria and is found in Staphylococci (Zinner and Mayer, 2015; Rossolini et al., 2017). Surpringsily, the variants *dfrA12*, *dfrA17*, and *dfrA5* showed higher percentages in the isolates from environmental sources compared with animal and human isolates, where the proportions were equal.

### Plasmid content

The plasmids represent the mobile genetic element (MGE) that was considered the principal vector for horizontal gene transfer (HGT) of the variants of *mcr-1* reported to date (Shen et al., 2016; Sun et al., 2018). Unfortunately, the sequences data were mainly based on short-read sequencing. This is because the identification of the specific location of the resistance genes is unclear because *de novo* assembly of reads from total genomic DNA does not allow the separation of the assemblies according their original location (plasmids or chromosome; Wang et al., 2021).

IncFIB, IncFII, IncI2, IncI1, IncFIC, IncFIA, IncX1, and IncX4 were the plasmids with the highest occurrence in animal, human, food, and environmental samples. Moreover, these plasmids have been reported to be associated with HGT for *mcr-1* variants and other ARGs (Ghasemian et al., 2018; Daza-Cardona et al., 2022). Geographically, there is an apparent homogeneity in the presence of the plasmids IncFIB, IncFII, and IncI2. However, in Europe, the occurrence of the plasmid IncI1 was notably higher in comparison with the other continents. The same trend was observed with the plasmid IncX4, with a considerable presence in isolates from western Asia and Europe compared to the rest of Asia and America. In addition, the plasmid incompatibility group IncN was more frequent in the southeast Asia region. IncHI2A and IncHI2 exhibited similar occurrences according to the geographical origin; however, these incompatibility groups were not present in environmental samples. IncHI1B and IncHI1A were present only in samples from America and Southeast Asia, and the higher incidence was in human samples. The presence of these plasmids was null in environmental isolates.

Conversely, IncA/C2 was the only plasmid absent in America, Europe and Africa but latent in Asia. This, in turn, had a low incidence in animal and human samples, being absent in food and environmental samples. Finally, the IncX2, IncX3 and Col plasmids were the only plasmids that were not present in Asia but only present in America and even there in small numbers. These in turn, were not present in food or environmental samples. The difference between IncX3 and IncX2 and Col was absent in animal samples.

Therefore, based on the information collected, it can be indicated that the highest diversity of plasmids was found in animal samples, followed by human, and environmental samples. In turn, Asia was the continent that led with the largest diversity and occurrence of these plasmids, followed by America and Europe.

## Conclusion

Whole-genome sequencing allows the scientific community to better understand the evolution and dissemination of antibiotic resistance worldwide. However, several questions still need to be answered. As noted above, the *E. coli* isolates that harbor *mcr-1* showed a wide plethora of ARGs, plasmid-types and virulence genes, demonstrating in most cases a multiresistance profile based on their genotypic information. It is essential to note the higher percentages of co-resistant ARGs detected in environmental samples of clinical relevance, which highlights the critical role of the environment in the AMR crisis. However, these results only represent the top of the iceberg, considering that WGS is not considered as part of the surveillance programs in the majority of countries. Additionally, it is remarkable how limited are the number of isolates from environmental origin and the geographical bias for the analysis of AMR worldwide. These findings highlight the relevance of analyzing the environmental settings as an integral part of the surveillance programs to understand the origins and dissemination of AMR.

## Author’s note

This article was developed through the Structured Operational Research and Training Initiative (SORT IT), a global partnership coordinated by TDR, the UNICEF, UNDP, World Bank, WHO Special Program for Research and Training in Tropical Diseases hosted at the World Health Organization. The specific SORT IT program that led to this research article included an implementation partnership of TDR and the Pan American Health Organization (PAHO), the WHO Country offices of Colombia and Ecuador; the Ministry of Health and Social Protection, Colombia; Food and Agriculture Organization, Sierra Leone; Sustainable Health Systems, Freetown, Sierra Leone; The Tuberculosis Research and Prevention Center Non-Governmental Organization, Armenia; The International Union Against Tuberculosis and Lung Diseases, Paris, France and South East Asia offices, India; Institute of Tropical Medicine, Antwerp, Belgium; Damien Foundation, Belgium; Indian Council of Medical Research-National Institute of Epidemiology; Jawaharlal Institute of Postgraduate Medical Education and Research (JIPMER); GMERS Medical College Gotri Vadodara Gujarat, India; India Medical College Baroda, Gujarat, India; Sri Manakula Vinayagar Medical College, India; Public Health, Ontario; The Universidade Federal de Ciencias de Saude de Porto Alegre, Brazil; Universidade de Brasilia, Brazil; Universidad de Concepcion, Chile, Universidad de los Andes, Colombia; Universidad Pontificia Bolivariana, Colombia; Universidad Pedagógica y Tecnológica de Colombia; The Central University of Ecuador and; California State University of Fullerton, United States; The Autonomous University of Yucatán, México.

## Data availability statement

The datasets presented in this study can be found in online repositories. The names of the repository/repositories and accession number(s) can be found in the article/Supplementary material.

## Author contributions

WC-C designed the investigation. KR, AM, JM, and CB-C performed the preliminary analyses of the data. WC-C, KR, AM, and JM performed genomic analysis, including genome assemblies, MLST, antibiotic resistance genes, plasmid and cgMLST analysis. MR, NO-G, TS, CD, and AH collaborated in the protocol designing according to Structured Operational Research and Training Initiative SORT IT (Partnership between TDR-UNICEF, UNDP, World Bank and WHO). WC-C, AH, NO-G, TS, CD, CB-C, and MR obtained the approbation from the Ethics Advisory Groups from PAHO and The Union. WC-C supervised the entire work. WC-C, AM, JM, and AH wrote the manuscript. All authors contributed to the article and approved the submitted version.

## Conflict of interest

The authors declare that the research was conducted in the absence of any commercial or financial relationships that could be construed as a potential conflict of interest.

## Publisher’s note

All claims expressed in this article are solely those of the authors and do not necessarily represent those of their affiliated organizations, or those of the publisher, the editors and the reviewers. Any product that may be evaluated in this article, or claim that may be made by its manufacturer, is not guaranteed or endorsed by the publisher.

## Footnotes

^1^www.real-statistics.com.
